# Systematic Development of Materials for Inviting Low Health-Literate Individuals to Participate in Preconception Counseling

**DOI:** 10.3390/ijerph16214223

**Published:** 2019-10-31

**Authors:** Laxsini Murugesu, Miriam E. Hopman, Sabine F. Van Voorst, Ageeth N. Rosman, Mirjam P. Fransen

**Affiliations:** 1 Amsterdam Public Health Research Institute, Department of Public Health, University of Amsterdam, Amsterdam UMC, 1105 AZ Amsterdam, The Netherlands; m.e.hopman@amsterdamumc.nl (M.E.H.); m.p.fransen@amsterdamumc.nl (M.P.F.); 2 Department of Obstetrics and Gynaecology, Erasmus University Medical Center, 3015 GD Rotterdam, The Netherlands; s.vanvoorst@erasmusmc.nl (S.F.V.V.); a.n.rosman@hr.nl (A.N.R.); 3 Department of Health Care Studies, Rotterdam University of Applied Sciences, 3015 EK Rotterdam, The Netherlands

**Keywords:** recruitment, health literacy, written invitation, preconception counseling

## Abstract

In this study we aimed to systematically analyze problems in the recruitment of women with low health literacy for preconception counseling and to adapt and evaluate written invitations for this group. In a problem analysis (stage 1) we used structured interviews (n = 72) to assess comprehension of the initial invitations, perception of perinatal risks, attitude and intention to participate in preconception counseling. These outcomes were used to adapt the invitation. The adapted flyer was pretested in interviews (n = 16) (stage 2) and evaluated in structured interviews among a new group of women (n = 67) (stage 3). Differences between women in stages 1 and 3 regarding comprehension, risk perception, attitude and intention to participate in counseling were analyzed by linear regression analysis and chi-square tests. Women in stage 3 (who read the adapted flyer) had a more positive attitude towards participation in preconception counselling and a better understanding of how to apply for a consultation than women in stage 1 (who read the initial invitations). No differences were found in intention to participate in preconception counseling and risk perception. Systematic adaptation of written invitations can improve the recruitment of low health-literate women for preconception counselling. Further research should gain insight into additional strategies to reach and inform this group.

## 1. Introduction

Poor perinatal outcomes remain a problem in Western countries, despite the development of perinatal care [1,2]. Up to 35% of adverse perinatal outcomes, such as preterm birth, small for gestational age and congenital disorders are preventable [3,4]. Interventions should start before conception to minimize the risks of adverse perinatal outcomes [1,4,5,6].

Preconception counseling (PCC) targeted at (future) parents can play a major role in reducing poor perinatal outcomes [6]. PCC includes health education and promotion. It aims to enable informed decision-making regarding conception and to assess and reduce preconception health risks and the risk on adverse perinatal outcomes [7,8].

Despite the benefits of PCC, barriers to implement this care still exist [9]. Such barriers include lack of accessibility to PCC, especially for those who need it most. Individuals with a lower socioeconomic status (SES) have the highest risk of adverse perinatal outcomes, but hardly participate in PCC [2,10,11,12]. They often have lower health literacy [13], i.e., skills to assess, understand, appraise, and apply health-related information to make judgments and decisions in everyday life concerning healthcare, disease prevention and health promotion to maintain or improve quality of life during the life course [14].

Low health literacy is associated with poorer use of healthcare services, including preventive care [15]. This also accounts for reproductive healthcare. Low health-literate individuals seek reproductive care less often, screen for sexually transmitted diseases less often, and initiate prenatal care later in pregnancy [16,17,18]. Previous research also showed that PCC is mostly used by women who live in higher SES neighborhoods [19].

Written letters or invitations are commonly used tools in healthcare to spread a message easily and reach the target population. However, written health-related information is often poorly understood by low health-literate individuals and often not tailored to their needs [20]. A review of evidence for document design interventions have shown that using for instance narratives or combining textual with visual characteristics could enhance comprehensibility [21,22].

This study was embedded in a Dutch nationwide program called ‘Healthy Pregnancy for All’ (HP4All). HP4All was initiated by the Erasmus Medical Center in specific municipalities with perinatal mortality and morbidity above the country’s average. Hereby, they indirectly targeted low SES neighborhoods. Women were recruited for PCC by invitational letters and referrals. General practitioners and midwives provided the counseling [23].

**Figure 1:**
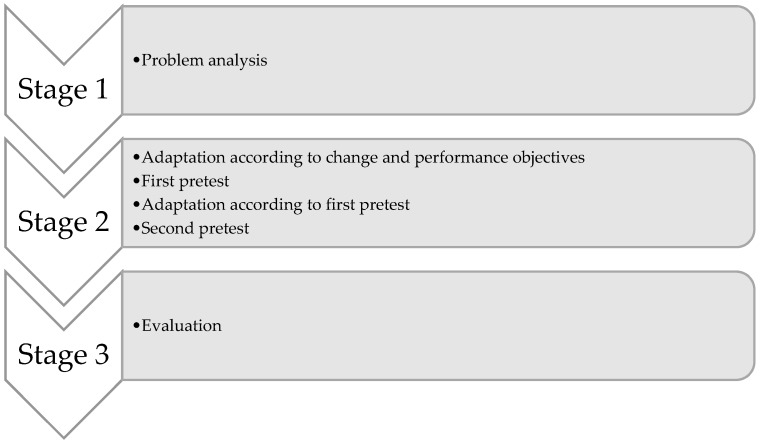
Three stages of systematic development of written invitational material.

We aimed to systematically analyze problems in the recruitment for PCC (stage 1), to adapt (stage 2) and evaluate (stage 3) written invitations about PCC for individuals with low health literacy (see Figure 1). Specific objectives were:

- to analyze comprehension of the HP4All invitational material, perception of perinatal risk, attitude towards PCC, intention to participate in PCC, and actual participation in PCC (stage 1);
- to adapt invitational material and assess acceptance, comprehensibility, and relevance of the adapted invitational material (stage 2);
- to evaluate the final version of the adapted invitation in a new group of low health-literate women (stage 3).

## 2. Materials and Methods

### 2.1. Study Design

For the systematic development of invitational material, we followed the intervention mapping approach in a non-experimental setting among low health-literate women who were eligible for PCC. Intervention mapping includes the following steps: a problem analysis, matrix of change objectives, selection of theory-based methods and practical strategies, program plan, adoption and implementation plan, and an evaluation plan [24]. The initial invitational materials of HP4All included: (I) an invitational letter from the municipal health service or municipality sent to all women residing in selected zip codes, (II) invitational letter from participating general practitioners, (III) referral by participating youth healthcare service, (IV) and a referral by a preconception health educator [23]. In a problem analysis, we reviewed the HP4All invitational referral and letter in structured interviews (stage 1). The outcomes were used to formulate performance and change objectives to adapt the invitational material, which was pretested in a first pretest. The invitation was then further adapted according to the outcomes of the first pretest. The final adaptions were pretested in a second pretest. (stage 2) and the final version of the invitation was evaluated in interviews among a new group of women (stage 3) (see Supplementary materials for the invitations used in Stage S1–S3).

### 2.2. Study Population and Recruitment

The study population consisted of low health-literate women between 18–42 years old who had a wish to become pregnant within the next five years. Hereby, we excluded women who certainly did not want to become pregnant (in the coming five years). Women who were unable to speak or read Dutch and women who were pregnant were also excluded for participation in the interview.

Women were recruited personally in waiting rooms of general practices, mother and child healthcare centers and youth healthcare centers in low SES neighborhoods.

Participants in stage 1 were recruited from a general practice and a youth healthcare center in Amsterdam that participated in the HP4All program (low SES neighborhood). In stage 2 two pretests were performed. Participants in the first pretest were recruited in municipal health centers for mother and child healthcare in Almere that also participated in the HP4All program. Participants in the second pretest were recruited from centers that did not participate in the HP4All program (a youth healthcare center, a primary school, and an intermediate vocational education school in Amsterdam, Almere, and Wageningen). Participants in stage 3 were recruited from the same locations as the second pretest. Participants of the second pretest and stage 3 were not recruited in HP4All areas, because HP4All was not available during this period.

Women were first asked whether they were interested to participate in a study on preconception care. When interested we informed them about the study and personally assessed health literacy with the Short Assessment of Health Literacy in Dutch (SAHL-D) (see below) [25]. We made an appointment for the interview when women scored low on this test.

The low health-literate women were invited for an interview of 30 min at a location that they preferred or for an interview by telephone. All participants were offered a gift voucher after completing the interview. Besides personal recruitment and gift vouchers to optimize recruitment, we made easy to read flyers, were flexible in time and location, and send reminders before the interviews took place.

### 2.3. Stage 1: Problem Analysis

In structured interviews we analyzed comprehension of the HP4All invitational referral and letter, perception of perinatal risk, attitude towards PCC, intention to participate in PCC, and actual participation in PCC. The interviews were guided by a questionnaire, and the items in this questionnaire are presented below.

#### 2.3.1. Conceptual Framework

The questionnaire for the structured interviews was based on Von Wagner’s framework for health literacy and health actions [18]. His framework proposes that health outcomes, e.g., preterm birth, are determined by the following actions: access and use of healthcare, such as PCC; patient–provider interactions; and management of health and illness. These actions are influenced by motivational determinants (e.g., knowledge, understanding, beliefs and attitudes), volitional determinants (e.g., information processing and decision making skills), and environmental determinants (e.g., availability or costs of PCC) [18].

#### 2.3.2. Background Characteristics

The following demographic characteristics were assessed: educational level [26], occupational status, relationship status, and ethnic background [27].

To assess the difficulty to understand Dutch the respondents, who were able to communicate in Dutch, were asked whether they had ever experienced difficulty understanding written or spoken Dutch [28].

Health literacy was assessed by the SAHL-D, a test based on word recognition and comprehension in the health domain. The cut-off points were defined in the validation study of the SAHL-D, those with <55 out of 66 correct answers were considered as low health-literate [25,29]. The cut-off point of 55 was estimated to correctly classify 74% of respondents with inadequate health literacy [25].

Wish to conceive was evaluated by asking whether the respondents contemplated pregnancy in upcoming five years.

Perinatal outcomes were evaluated by asking respondents whether they had been pregnant before, and if so, whether they had ever experienced unplanned pregnancy and/or problems during a previous pregnancy.

Awareness of PCC was explored by a close-ended and an open-ended question, namely ‘Have you heard about PCC?’ and ‘If so, who told you about it?’ or ‘If so, where did you hear or read about it?’ Answers were categorized within the predefined sources of information, such as ‘The general practitioner told me about it’ or ‘I read about it in the newspaper’. The respondents in the problem analysis group were also asked whether they received a HP4All invitation.

#### 2.3.3. Main Outcome Measures

Comprehension of the invitational material was subjectively and objectively measured after respondents read the material. Subjective comprehension was assessed by the perceived difficulty of comprehending the text. Response options ranged from 1 ‘very easy to understand’ to 5 ‘very difficult to understand’. Objective comprehension was assessed by four questions with multiple answer options on the target audience, aim, content, and application procedure of PCC.

Perception of perinatal risk was assessed by the following statement on a five-point Likert scale (ranging from 1 ‘strongly agree’ to 5 ‘strongly disagree’) ‘I probably won’t experience any problems during pregnancy.’.

Attitude towards PCC was assessed by asking respondents to rate participation in PCC as good versus bad, comforting versus scary, important versus unimportant, pleasant versus unpleasant, useful versus useless and embarrassing versus something to be proud of. We used a five-point Likert scale for the items in the attitudes towards PCC measure. The scale ranged from 6 to 30 [30,31]. For analysis, the total score was divided by the amount of items, which was six (Cronbach’s alpha = 0.77).

Intention to participate in PCC was assessed by asking respondents to rate the likelihood of their participation in PCC before their (next) pregnancy on a five-point Likert scale ranging from 1 ‘extremely unlikely’ to 5 ‘extremely likely’.

Actual participation in PCC was assessed by telephone a few weeks after the interview. We only called women who had a wish to conceive within two years, because PCC would be most relevant to them. We called women within a few weeks after the interview since PCC was only available in stage 1, and to exclude other factors that could play a role in whether or not to participate in PCC.

### 2.4. Stage 2: Adaptation of Material and Pretest

#### 2.4.1. Formulating Performance and Change Objectives Based on Outcomes Problem Analysis (Stage 1)

Based on the outcomes of the problem analysis (explained in detail in the results section), we defined the following performance objectives for adaptation of the materials:

- Is aware of written invitation for PCC
- Makes an informed decision whether (or not) to participate in PCC
- Participates in PCC

Change objectives were formulated by crossing these performance objectives with determinants that were also derived from the problem analysis (stage 1): risk perception, knowledge level, attitude, and coping skills (see Table 1: Performance and change objectives). For each change objective, we matched theory-informed intervention methods. An intervention method is a theoretically and empirically supported process for effective behavior changes [24]. These theoretical intervention methods were translated into practical intervention strategies to adapt the invitational material. For example, for the change objective ‘Is aware of the fact that she belongs to a high risk group’, we selected the theoretical method, ‘modeling’, and practical strategy: ‘using narratives’. An overview of all theoretical models with reference to the methods and accompanying practical strategies are presented in Table 2: Theoretical models, methods and strategies.

#### 2.4.2. Adaptation and Pretesting of Invitational Material

Based on the practical strategies (Table 2), the following adaptations were made to the initial letter: a case model story with a narrative was added to inform women about the advantages and disadvantages related to participation in PCC. Within the case model story it was explained how women could make an appointment and deal with practical problems, like arranging a baby sitter. In the story, norms and values were expressed to help women imagine what it is like to experience the physical, emotional and social effects of participating in PCC. For example, although the representative in the case model first mentions that she doubts whether she should participate in PCC, she eventually participates because she considered preparing herself for pregnancy as very important. The title “Invitation for preconception counseling” was added to strengthen the association with an invitation. Finally, the text was adapted by using shorter sentences, clear subheadings, and less complex words.

These first adaptations were pretested in interviews, which aimed to assess acceptance, comprehensibility, and relevance of the invitational material. We assessed specifically to what extent the material was appealing for them, by asking what they thought of the layout. We also asked women if they considered PCC to be relevant for themselves after reading the material, by asking whether they would be interested in participating in PCC in the future and why. Objective and subjective comprehension was assessed by the same items as in stage 1.

Based on this first pretest, the letter was adapted by the Center for Media and Health, Gouda, Netherlands (https://www.media-health.nl/). Firstly, the letter was adapted into a flyer to make the invitation more appealing and reduce the amount of text. Secondly, the aim, procedure, and advantages of PCC were stated more condensed by using shorter sentences. We used a picture of a representative with a suitable quote to reduce the text, but to keep a narrative story which can be recognized in the picture and to make the message more appealing [32]. Finally, the title ‘Visit preconception counseling to give your child a healthy start’ was added to strengthen the association with an invitation and to activate participation. The adapted version was again pretested among a new group of women with low health literacy. Besides the questions of the first pretest, we also obtained women’s preferences regarding three representatives and quotes.

### 2.5. Stage 3: Evaluation of Adapted Material

Stage 3 aimed to evaluate the final version of the invitation in a new group of low health-literate women. Data from this evaluation were compared with data derived from the problem analysis group. During the interview, the same procedure and questionnaire were used as during the problem analysis (measures are described in detail above). The only difference was the invitational material which was evaluated and we did not assess actual participation among the evaluation group.

### 2.6. Analyses

#### 2.6.1. Stage 1

Descriptive statistics were used to summarize the main outcome measures of stage 1. We assessed Cronbach’s alpha for all item-measures. All items scored above 0.7, except the objective comprehension item ‘application procedure of PCC’. This item was assessed separately (n, %).

#### 2.6.2. Stage 2

Descriptive statistics were used to summarize background characteristics of the first and second pretest for the overall pretest group. Open answers on acceptance, the extent the material was appealing and relevant for women, and reasons for (not) participating in PCC were summarized per respondent. The preferences regarding three representatives and quotes were counted. The representative and quote that was preferred by most of the respondents was chosen for the final version of the invitational material.

#### 2.6.3. Stage 3

Descriptive statistics were used to summarize the main outcome measures of stage 3. Differences in the main outcome measures between the women in the problem analysis and women in the evaluation group were analyzed by linear regression analyses. Confounding was checked for the outcomes which had significant *p*-values in the raw model, i.e., only for attitude towards PCC and objective comprehension. Significant results were adjusted for potential confounders, i.e., health literacy and occupational status. Health literacy correlated with age, educational level, ethnic background, difficulty understanding Dutch and perinatal experiences related variables. The covariate was considered as a confounder and left in the model, when it changed the variation in score by 10% or more. The objective comprehension item ‘application procedure of PCC’ was analyzed by a chi-square test, since everyone in the evaluation group answered correctly. Data were analyzed using the SPSS Statistics 23 software.

### 2.7. Ethics Approval and Consent to Participate

The Medical Ethics Committee of the Academic Medical Center of the University of Amsterdam approved this study in 2013. Written consent to participate in the interviews was obtained from all respondents, so that participation was entirely voluntary. For telephone interviews we obtained oral consent.

## 3. Results

### 3.1. Response and Background Characteristics

**Figure 2:**
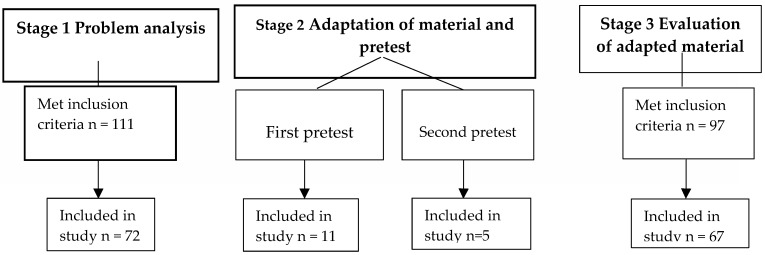
Inclusion study population.

In total, 224 women met the inclusion criteria, 155 of them participated in the structured interviews of stage 1, 2, and 3 (Figure 2). The HP4All invitations were reviewed in the problem analysis (stage 1) by 72 respondents (See Table 3 for characteristics). The adapted invitation was tested by 11 women in the first pretest and by 5 women in the second pretest (stage 2). The final version of the invitational material was evaluated in a new group of 67 respondents (stage 3).

The problem analysis (stage 1) and evaluation group (stage 3) significantly differed for educational level, ethnic background, difficulty in understanding Dutch, perinatal experiences and for wish to conceive. No differences were found for relationship and occupational status. Although all respondents were able to communicate in Dutch, 5% of the respondents in the evaluation group still had difficulty understanding the Dutch language. This is relatively low compared to the problem analysis group. The problem analysis group more often experienced a pregnancy before and also experienced more unplanned pregnancies than the evaluation group (Table 3).

Out of 72 respondents from stage 1 that lived in municipalities where HP4All invitation materials were disseminated, 31% reported having heard or read about the concept of PCC and 17% recalled seeing the invitation material. From the respondents in stage 3 who were recruited in other centers, 13 respondents (19%) were aware of PCC.

### 3.2. Outcomes Stage 1: Problem Analysis

#### 3.2.1. Subjective and Objective Comprehension

After reading the HP4All invitational material, most respondents rated these materials as easy or very easy to comprehend (Table 4). They scored highest on comprehension on the target population of PCC. The lowest comprehension scores were obtained for how one could make an appointment for PCC.

#### 3.2.2. Risk Perception, Attitude and Intention

Respondents generally agreed with the statement “I probably won’t experience any problems during pregnancy” (mean score 2.6, range 2–4). This indicates that they perceived a low risk of perinatal problems.

Most respondents had a positive attitude towards PCC (mean score 3.7, range 2–4). In total 50% of the respondents intended to participate in PCC (mean score 3.1, range 1–5).

Within three months after the interview, we were able to contact 24 out of 30 respondents who had reported to have a wish to conceive in the next two years. None of these respondents had made an appointment for PCC.

### 3.3. Outcomes Stage 2: Pretest and Adaptation of Material

The first pretest of the adapted letter and referral showed that the respondents (n = 11) had a positive attitude towards PCC. See Table 5 for background characteristics of the respondents. They recognized themselves in the narrative story, seemed to understand the aim and content of the invitation, and were aware of the advantages and disadvantages of PCC. Although most women had a positive attitude towards PCC, they did not have the intention to participate. The letter did not seem to be appealing to them, because they did not feel they belonged to the target population for PCC. They explained that they only read the letter since we asked them to for the interview, but in real life they would not read this large amount of text.

In the second pretest the adapted material consisted of a flyer with a small amount of text, shorter sentences, simple words, and a picture and quote instead of a narrative story. The results of this pretest showed that women (n = 5) had a preference for the picture of a young woman that quoted ‘We are considering having a baby and want to know as much as possible’. Women explained that they liked the fact that she prepared for her pregnancy at a young age by looking for information. Despite the fact that the content of the adapted material was shortened, women did not seem to miss information since all five women answered the comprehension questions about the aim and procedure of counseling correctly. Three women responded that they would participate in PCC if they were invited. The other two women said that they would not participate in PCC. One of them mentioned that she would participate only if she experienced problems conceiving.

### 3.4. Outcomes Stage 3: Evaluation of Adapted Material

#### 3.4.1. Differences in Comprehension of Invitational Material

Almost all women (96%) who participated in the evaluation of the adapted materials (n = 67) rated the adapted flyer as either easy or very easy. In the problem analysis group (n = 72) 93% of the women rated the initial letter and referral as (very) easy. Linear regression analysis showed that this difference in subjective comprehension was non-significant between both groups (Table 6).

In total, 64% of the women in the problem analysis group understood the application procedure correctly, while almost all women in the evaluation group answered this question correctly (97%, 1 missing). The chi-square test showed that the difference in objective comprehension between both groups regarding the application procedure was significant (*p* < 0.0001; 95% confidence interval (CI): 1.68–2.54). The evaluation group also scored higher than the problem analysis group on the remaining items of objective comprehension (beta: 0.09, 95% CI: −0.13–0.31) (Table 6).

#### 3.4.2. Differences in Risk Perception, Attitude and Intention

Participants in the problem analysis and evaluation both scored relatively low on perinatal risk perception (Table 7). Linear regression analysis showed that the evaluation group had a higher perception of perinatal risk than the problem analysis group (beta: 0.14, 95% CI: −0.19–0.47).

Both groups scored high on attitude towards PCC, indicating that both groups had a positive attitude towards participation in PCC. Linear regression analysis showed that the women in the evaluation had a significantly more positive attitude towards PCC than the women in the problem analysis group (Table 7).

For intention to participate in PCC both groups scored around 3.0 (scale 1 to 4), which meant that they rated themselves as ‘maybe willing to participate in PCC’. The linear regression analysis showed a negative regression coefficient, indicating that women in the problem analysis group were more likely to participate in PCC compared to women in the evaluation group (Table 5).

## 4. Discussion

### 4.1. Main Findings

The problem analysis (stage 1) showed that low health-literate women were generally unaware of the invitations that were provided by the HP4All program. Most respondents rated the HP4All invitational material as easy or very easy to comprehend. However, women scored low on how one could make an appointment for PCC. Despite a positive attitude towards PCC, only half of the women intended to participate in PCC, which indicates that women did not make informed choices. Women also had a low perceived risk of perinatal problems. The first pretest (stage 2) showed that the invitational material contained a lengthy text, which the women found undesirable. They also did not consider PCC as relevant to them. After the second adaptations of the invitational material (stage 2), women found the material more appealing and accessible, since it contained less text and a picture of someone that they could identify with. Stage 3 (evaluation stage) showed that the adapted invitation resulted in a higher attitude towards participation in PCC, but women did not feel it was relevant to them personally. Women had a significantly better understanding for how to apply for PCC, which optimizes informed decision-making. Adaptation of the materials did not lead to significant changes in the other comprehension questions about the invitational material, their perception of perinatal risk and intention to participate in PCC.

### 4.2. Discussion of Main Findings

The problem analysis (stage 1) showed that respondents had low awareness of the availability of PCC, which is in accordance with other studies reporting that individuals with low health literacy participate less in preventive care [18]. A possible explanation for respondents’ low awareness, even if the respondents lived in an area where HP4All invitations for counselling were distributed, could be that low health-literate individuals generally do not search for information if they do not have any problems or needs. Another explanation could be that they have seen the invitational material, but did not read it, for example because the text was too long or the layout of the invitation was not appealing.

The problem analysis also showed that women with low health literacy did not consider themselves to be at risk of perinatal problems. However, previous research shows that women with low health literacy have greater risk on adverse perinatal outcomes than women with higher levels of health literacy [16]. Incorrect risk perception is a common problem in prevention, and seems to be particularly problematic in individuals with low health literacy [33].

During the pretests (stage 2), women also mentioned that PCC is an important preventive measure, but that they did not feel it was relevant to them personally. This is reflected in a relatively low intention and participation, despite a positive attitude towards PCC. Mazza et al. (2010) also found dissonance between women’s positive motivation to improve their health in preparation for pregnancy and resisting participation in PCC [11]. Our respondents felt that PCC is restricted to women or couples contemplating pregnancy with complex risk factors, which they thought they did not have. Poels et al. (2016) have shown that low-risk perception is one of the most important barriers for participation in preconception care [34].

Our adaptation in stage 2 consisted of shortening the amount of text, using shorter sentences, simple words, and a picture and quote instead of a narrative story. Previous research on patient education materials has shown that these strategies improve readability [35]. Meppelink et al. (2015) showed that, for instance, illustrated messages were especially effective among low health-literate individuals [36].

The evaluation of the adapted materials (stage 3) indicated that even when the invitational material was shortened, the content is still comprehensible. The fact that we did not find differences in comprehension of the invitational material between both groups was expected, because knowledge was already high (ceiling effect), probably because HP4All already paid a lot of attention to clear use of language, short sentences and easy to read words. The high scores on objective comprehension contradicted with other studies on comprehension of written materials, which reported less positive results. A study among female attendees of a family planning clinic found that women with low health literacy had difficulty understanding leaflets on contraception [37]. Other studies confirmed this finding within different fields of healthcare [38,39].

### 4.3. Strengths and Limitations

A strength of this study is that we used face-to-face interviews or interviews by telephone. This ensured that all respondents understood the questions correctly and were able to ask any questions when in doubt.

Another strength is that low health-literate women were included in all stages of this study. This is important since the uptake of PCC is especially low among this group. Furthermore, we used intervention mapping which implies that adaptions of the invitational material were theory- and evidence-based [24]. We conducted the first four steps of intervention mapping, including a problem analysis, formulating change and performance objectives, selecting theory, methods and strategies that match the objectives, and we adapted the material accordingly. Since HP4All PCC was not provided in the evaluation stage of our study, we were not able to complete the last two steps of intervention mapping: i.e., assess the implementation and sustainability of the adaptations in real-life contexts, and conduct an effect and process evaluation [24]. We were also not able to measure actual participation in stage 3, since PCC provided by HP4All was not available anymore during this period. Even though stage 3 was conducted outside the context of HP4All, these outcomes are still essential to improve accessibility of PCC. The outcomes can be used to develop invitational materials for PCC for women with low health literacy. Finally, the fact that HP4All already paid a lot of attention to make the text easy to understand led to a ceiling effect; therefore, we only found one significant difference in comprehension outcomes (understanding how to apply for PCC).

Written invitational material was a suitable strategy for the HP4All program to reach a specific group of women with perinatal mortality and morbidity above the country’s average. However, low awareness of PCC and low risk perception suggest that tailored recruitment strategies to the needs of low health-literate women require more effort. We were not able to test other recruitment strategies, since we were restricted to the written material of HP4All. Nevertheless, this systematic approach can be used by others to improve recruitment of individuals with low health literacy in other contexts of (preventive) healthcare.

### 4.4. Implications for Further Research and Practice

Systematic adaptation of written materials improved understanding on how to apply for PCC, which optimizes informed decision-making, and attitudes towards PCC among low health-literate individuals, but did not improve the intention to participate in PCC and risk perception. Other factors also play a role, including considerations to participate in PCC, subjective norms, self-efficacy, awareness, and overall knowledge about preconception care [40]. These factors should be taken into account. Since written invitations have various limitations and are not sufficient to reach and inform low health-literate individuals, further research should gain insight into different additional recruitment strategies within and outside the context of PCC. To raise awareness for PCC, young women could be attended on their personal risks by their GP when other preconception topics are discussed (e.g., anticonception). Another option is to actively approach women with a wish to conceive by strategies such us entertainment education and social media. However, to improve risk perception PCC only would not be enough, and further research is required to investigate which strategies are suitable to improve risk perception among women with low health literacy.

## 5. Conclusions

Systematic adaptation of written invitations can improve the recruitment of low health-literate women for preconception counselling. The adapted initial invitational material seemed to have a positive impact on attitude but did not improve risk perception and the intention to participate in PCC. Further research should gain insights into additional strategies to reach and inform women with low health literacy about PCC and to increase overall awareness of (the importance of) PCC.

## Figures and Tables

**Table 1:** Performance and change objectives.

Performance Objectives	Determinants			
	Risk Perception	Knowledge Level	Attitude	Coping Skills
Is aware of written invitation for PCC.		Knows that she is invited by the written invitation material of PCC.		
Makes an informed decision whether (or not) to participate in PCC.	Is aware of the fact that she belongs to a high risk group.	Knows what PCC entails.	Has a positive attitude towards participation in PCC. The decision whether or not to participate in PCC corresponds with attitude. Weighs the advantages and disadvantages and assigns value to these advantages and disadvantages.	
Participates in PCC.				Is able to cope with practical barriers.

**Table 2:** Theoretical models, methods and strategies.

Performance Objectives	Change Objectives	Theoretical Models	Methods	Strategy
Is aware of written invitation for PCC.	Knows that she is invited by the written invitation material of PCC.	Dual-process model (William James, 1890). Systemic functional linguistics (Halliday et al., 1994).	Stimulating women’s intuitive and automatic cognitive processes. Adjusting text attributes.	Strengthen association letter with an invitation. Adjusting text attributes.
Makes an informed decision whether (or not) to participate in PCC.	Is aware of the fact that she belongs to a high-risk group.	Social Cognitive Theory, (Bandura, 1986.) Theory of Heuristics (Herbert Simon, 1957). Systemic functional linguistics (Halliday et al., 1994).	Modeling. Adjusting text attributes.	Role model story. Using narratives. Adjusting text attributes.
Makes an informed decision whether (or not) to participate in PCC.	Knows what PCC entails.	Dual process model (William James, 1890). Systemic functional linguistics (Halliday et al., 1994).	Stimulating women’s working memory and deliberative, logical and analytical cognitive processes. Adjusting text attributes.	Role model story.Using narratives. Adjusting text attributes.
Makes an informed decision whether (or not) to participate in PCC.	Weighs the advantages and disadvantages and assigns value to these advantages and disadvantages.	Social Cognitive Theory, (Bandura 1986). Theory of Heuristics (Herbert Simon, 1957). Systemic functional linguistics (Halliday et al., 1994). Dual-process model (William James, 1890).	Modelling. Adjusting text attributes. Using International Patient Decision Aids Standards (IPDAS) criteria.	Role model story.Adjusting text attributes. Apply IPDAS criteria in narrative.
Makes an informed decision whether (or not) to participate in PCC.	The decision whether or not to participate in PCC corresponds with attitude.	Dual process model (William James, 1890). Systemic functional linguistics (Halliday et al., 1994).	Using IPDAS criteria. Adjusting text attributes.	Apply IPDAS criteria in narrative. Adjusting text attributes.
Women participate in PCC.	Is able to cope with practical barriers.	Social Cognitive Theory, (Bandura 1986). Theory of Heuristics (Herbert Simon, 1957). Systemic functional linguistics (Halliday et al., 1994).	Modelling. Adjusting text attributes.	Clearly stating how women should deal with practical problems. Adjusting text attributes.

**Table 3:** Background characteristics stage 1 and 3 (n = 139).

Background Characteristics	Stage 1 (n = 72)		Stage 3 (n = 67)		Stage 1 and 3 (n = 139)	
	**Mean (SD; Range)**	**N (%)**	**Mean (SD; Range)**	**N (%)**	**Mean (SD; Range)**	**N (%)**
Age (years)			29 (7; 18–42)		30 (6; 18–42)	
Educational level ^1^						
Low		**9 (13)**		**1 (2)**		**10 (7)**
Intermediate		**37 (51)**		**44 (66)**		**81 (58)**
High		26 (36)		22 (33)		48 (35)
Occupational status						
Employed		37 (51)		39 (58)		76 (55)
Student		13 (18)		17 (25)		30 (22)
Unemployed		22 (31)		11 (16)		33 (24)
Ethnic background ^2^						
Dutch		**13 (18)**		**41 (61)**		**54 (39)**
Other western (non-Dutch)		**13 (18)**		**10 (15)**		**23 (17)**
Non-western		**46 (64)**		**16 (24)**		**62 (45)**
Health literacy score	**35 (13; 9–53)**		**47 (7; 20–54)**		**41 (12; 9–54)**	
Difficulty understanding Dutch						
Sometimes		**29 (40)**		**3 (5)**		**32 (23)**
Never		**42 (58)**		**64 (96)**		**106 (76)**
Relationship status						
Married/Living together with partner		46 (64)		43 (64)		89 (64)
Single/Not living together with partner		26 (36)		24 (36)		50 (36)
Perinatal experiences						
Was pregnant before		**66 (92)**		**45 (67)**		**111 (80)**
Ever had an unplanned pregnancy		**38 (53)**		**16 (24)**		**54 (39)**
Ever had problems in previous pregnancy		35 (49)		26 (39)		61 (44)
Wish to conceive ^3^						
In next 2 years		**30 (42)**		**11 (16)**		**41 (30)**
In 2–5 years		31 (43)		30 (45)		61 (44)
Undecided		**11 (15)**		**26 (39)**		**37 (27)**

^1^ Significant differences (*p* < 0.05) between both groups are marked in bold. A significant difference was found between stage 1 and stage 3 for low and intermediate educational level *p* = 0.024 (95% confidence interval (CI) −0.084–(−0.04)). ^2^ A significant difference was found between stage 1 and stage 3 for Dutch and other Western *p* = 0.013 (95% CI 0.05–0.60) and Dutch and non-Western *p* < 0.001 (95% CI 0.30–0.70). ^3^ A significant difference was found between stage 1 and stage 3 for undecided and in next 2 years *p* < 0.001 (95% CI 0.17–0.70).

**Table 4:** Comprehension of HP4All invitation (n = 72).

Comprehension of HP4All Invitation	N (%)	Correct N (%)
**Subjective comprehension letter**		
Easy/very easy	67 (93)	
Neutral	5 (7)	
Difficult/very difficult		
**Subjective comprehension referral**		
Easy/very easy	64 (89)	
Neutral	8 (11)	
Difficult/very difficult		
**Objective comprehension letter**		
Target audience		67 (93)
Content counseling		65 (90)
Aim counseling		53 (74)
Application procedure		46 (64)
**Objective comprehension referral**		
Content counseling		64 (89)
Aim counseling		70 (97)
Application procedure		63 (88)

**Table 5:** Background characteristics stage 2 (n = 16).

Background Characteristics	First Pretest (n = 11)	Second Pretest (n = 5)
Age mean (range)	30 (19–39)	32 (28–37)
**Ethnic background**		
Dutch	7 (64%)	1 (20%)
Western (other than Dutch)	0 (0%)	1 (20%)
Non-Western	4 (32%)	3 (60%)
**Number children**		
mean (range)	2 (1–7)	1 (0–2)

**Table 6:** Differences in comprehension between stage 1 and 3 (n = 139).

Comprehension	Stage 1 (n = 72)	Stage 3 (n = 67)	Difference Stage 1 and 3
	N (%)	N (%)	Beta (95% CI)
Subjective comprehension letter *			0.05 (−0.02–0.12)
Easy/very easy	67 (93)	64 (96)	
Neutral	5 (7)	1 (2)	
Difficult/very difficult	0 (0)	0 (0)	
**Objective comprehension letter**			0.09 (−0.13–0.31) ***
Correct answers ‘target audience’	67 (93)	63 (94)	
Correct answers ‘content counseling’	65 (90)	65 (97)	
Correct answers ‘aim ‘counseling	53 (74)	62 (93)	
Correct answers ‘application procedure’ **	46 (64)	65 (97)	

* 2 missings; ** Significant difference in ‘application procedure’ (*p* > 0.0001) between stage 1 and stage 3; *** Adjusted for health literacy as a confounder.

**Table 7:** Differences between stage 1 and 3 in risk perception, attitude, and intention.

Risk Perception, Attitude and Intention	Stage 1 (n = 72)	Stage 3 (n = 67)	Difference Stage 1 and 3
	Mean (SD; range)	Mean (SD; range)	Beta (95% CI)
**Risk perception**	2.6 (0.9; 2–4)	2.7 (1.1; 1–5)	0.14 (−0.19–0.47)
**Attitude towards preconception counseling**	**3.7 (0.4; 2–4)**	**4.3 (0.6; 3–5)**	0.71 (0.48–0.93) *
**Intention to participate**	3.1 (1,5; 1–5)	3.0 (1.1; 1–5)	−0.13 (−0.57–0.31)

Significant differences (*p* < 0.05) between stage 1 and stage 3 are marked in bold; * Adjusted for health literacy as confounder.

## Supplementary Materials

The following are available online at https://www.mdpi.com/xxx, Stage S1: HP4All Invitational Letter from Participating General Practitioners; Stage S2: HP4All Referral; Stage S3: First Pretest; Stage S4: Second Pretest (Final Invitational Flyer).

## Abbreviations

PCC: preconception counseling includes health education and promotion, and is provided before a first pregnancy or between pregnancies; HP4All: Healthy Pregnancy for All is a Dutch nationwide program initiated by Erasmus MC in specific municipalities with perinatal mortality and morbidity above the country’s average; SAHL-D: Short Assessment of Health Literacy in Dutch; IPDAS: International Patient Decision Aids Standards.
