# Supplementary material for: Systematic Development of Materials for Inviting Low Health-Literate Individuals to Participate in Preconception Counseling
Source: Int J Environ Res Public Health. 2019 Oct 31;16(21):4223. doi: 10.3390/ijerph16214223 (PMC6862136; doi:10.3390/ijerph16214223)

International Journal of
Environmental Research and Public Health

MDPI

Supplementary

Stage S1: Stage 1: HP4All Invitational Letter from Participating General Practitioners

Geachte mevrouw,

Met deze brief brengen wij u op de hoogte van een nieuw project in onze gemeente, waar onze praktijk aan meedoet. Met het project 'Healthy Pregnancy 4 All' (een gezonde zwangerschap voor iedereen) bieden wij vrouwen of stellen met een kinderwens persoonlijk advies aan over gezond zwanger worden. Dit is uniek in Nederland en daarom sturen wij vrouwen tussen 18-42 jaar uit onze praktijk deze brief met uitleg over het kinderwensspreekuur.

Wij hebben geprobeerd zo veel mogelijk rekening te houden met persoonlijke omstandigheden. Het kan zijn dat dit toch niet gelukt is en deze brief ongepast of pijnlijk voor u is, bijvoorbeeld omdat zwanger worden niet wil lukken of niet mogelijk is. Daarvoor bieden wij u onze excuses aan. Het is niet de bedoeling om u met deze brief te kwetsen.

Waarom doen we mee aan dit project?

De babysterfte in Nederland is hoog en te veel baby's hebben een slechte start. Het Erasmus MC uit Rotterdam heeft een project ontwikkeld om de gezondheid van vrouwen voorafgaand aan de zwangerschap te verbeteren en eventuele risico's op te sporen. Hoe gezonder er aan een zwangerschap begonnen wordt, hoe groter de kans is op het krijgen van een gezonde baby. Het project 'Healthy Pregnancy 4 All' wordt in de wijk opgezet in samenwerking met gemeente, het Centrum voor Jeugd en Gezin (consultatiebureau/GGD) verloskundigen en huisartsen.

Wat houdt het kinderwensspreekuur in?

Op dit spreekuur krijgen vrouwen of stellen met een kinderwens, een persoonlijk advies over hoe zij zich zo goed mogelijk op een gezonde zwangerschap kunnen voorbereiden. In de eerste maanden van de zwangerschap, waarbij u vaak zelf nog niet eens weet dat u zwanger bent, is de baby het meest kwetsbaar: de organen van de baby worden dan al aangelegd. Daarom is het belangrijk om op deze eerste fase van de zwangerschap goed voorbereid te zijn. Verder kunt u vragen stellen over uw gezondheid, vruchtbaarheid en zwanger worden.

Bent u geïnteresseerd in het kinderwensspreekuur? Maak dan een afspraak!

U kunt contact opnemen met de assistente om een afspraak te maken. Vermeld even dat het gaat om een kinderwensspreekuur. Aan het project is een onderzoek verbonden. Als u een afspraak maakt wordt u gevraagd of u bereid bent hieraan deel te nemen. Dit is echter niet verplicht.

Met vriendelijke groet uw huisarts

Denk je na over zwanger worden?

Geef je kind een gezonde start en kom naar het kinderwensspreekuur!
Een gezonde zwangerschap vergroot de kans op een gezonde baby. Daarom is het voordat je zwanger bent al belangrijk om gezond te leven. Maar hoe weet je waar je op moet letten? Wat je beter wel en niet kunt doen? Of misschien heb je vragen over vruchtbaarheid. Voor antwoord op alle vragen over gezond zwanger worden, kun je naar het kinderwensspreekuur.

Kinderwensspreekuur
De kinderwensspreekuren worden gegeven door verloskundigen en huisartsen. Zij geven informatie en adviezen over zwanger worden en over een gezonde zwangerschap. Ze geven uitleg over de vruchtbare dagen van de maand en ze vertellen je waarom het goed is om foliumzuur te gebruiken. En dat je beter niet kunt roken. Maar ook wordt gekeken naar je gezondheid en of je baby een risico loopt door erfelijke ziektes. En soms is het beter als een gynaecoloog of andere medisch specialist in het ziekenhuis je begeleidt. Dan word je doorverwezen. Want het gaat om de gezondheid van jou en je baby.

Voor wie?
Iedereen die denkt aan zwanger worden, kan naar het kinderwensspreekuur. Al voordat je zwanger bent, kun je hier met je vragen naartoe.

Dus als je nog anticonceptiemiddelen gebruikt en er over denkt om daarmee te stoppen.

Waar kun je terecht?
Een verloskundige of huisarts bij jou in de buurt doet mee met het landelijke project Healthy Pregnancy 4 All. Onderaan deze flyer staat bij wie je een afspraak kunt maken voor het kinderwensspreekuur.

Waarom is het nodig?
Veel baby's worden te vroeg geboren of hun geboortegewicht is te laag. Met de kinderwensspreekuren willen we hier samen iets aan doen. Want een gezonde start van de zwangerschap vergroot de kans op een gezonde baby.

Meer informatie?
Bel voor meer informatie over de kinderwensspreekuren naar de huisarts of verloskundige die op deze flyer staat. En kijk voor meer informatie ook eens op **www.zwangerwijzer.nl**

Bel voor meer informatie en het maken van een afspraak voor het kinderwensspreekuur naar:

Huisarts A. van Beek
Gezondheidsstraat 1
1234 AB Amsterdam
020-123456

Stage S3: Stage 2: First Pretest

UITNODIGING KINDERWENSSPREEKUUR

Geachte mevrouw,

In Nederland worden veel baby's te vroeg geboren of hun geboortegewicht is te laag. Een gezonde zwangerschap geeft meer kans op een gezonde baby. Onze praktijk doet daarom mee aan het project 'Healthy Pregnancy 4 All' (een gezonde zwangerschap voor iedereen). Alle vrouwen tussen de 18-42 jaar krijgen deze uitnodiging voor het kinderwensspreekuur.

Wij hebben geprobeerd zo veel mogelijk rekening te houden met persoonlijke omstandigheden. Het kan zijn dat dit toch niet gelukt is en deze brief ongepast of pijnlijk voor u is. Bijvoorbeeld omdat zwanger worden niet wil lukken of niet mogelijk is. Daarvoor bieden wij u onze excuses aan. Het is niet de bedoeling om u met deze brief te kwetsen.

Wat houdt het kinderwensspreekuur in?

In het kinderwensspreekuur krijgt u van uw huisarts adviezen over zwanger worden en over een gezonde zwangerschap. Voordat u zwanger bent is het belangrijk om gezond te eten, voldoende te bewegen, niet te roken en geen alcohol te gebruiken. Maar hoe weet u nou waar u op moet letten? Wat u beter wel en wat u niet kunt doen? Of misschien heeft u wel vragen over vruchtbaarheid.

Voor wie?

Het kinderwensspreekuur is voor iedereen die denkt aan zwanger worden. Al voordat u zwanger bent kunt u met al uw vragen hier naartoe.

Hoe beslissen?

Denkt u rustig na, bedenk voordelen en nadelen, en praat er over met anderen. Hieronder leest u het verhaal van Sara.

"Hallo ik ben Sara en ik ben 27 jaar oud. Samen met mijn vriend heb ik een dochter van 2 en het lijkt me heel leuk om weer zwanger te worden. Laatst kreeg ik een uitnodiging voor het kinderwensspreekuur in de brievenbus. Ik twijfelde eerst een beetje, want mijn vriend en ik waren nog niet echt bezig met zwanger worden. Mijn eerste zwangerschap was goed gegaan, dus ik wist niet of het kinderwensspreekuur wel voor mij bedoeld was. Ook wilde ik mijn dochter niet meenemen naar het spreekuur, maar is het lastig voor mij om een oppas te regelen.
Maar ik vind het belangrijker om me goed voor te bereiden op een volgende zwangerschap, daarom wilde ik toch graag gaan. Mijn vriend vond het ook belangrijk. Ik heb de assistente van onze huisarts gebeld om een afspraak te maken voor het kinderwensspreekuur. De buurvrouw heeft op mijn dochtertje gepast.

We hebben ongeveer een half uur met de huisarts gepraat. Ik heb informatie gekregen en vragen kunnen stellen over foliumzuur, roken, alcohol en medicijnen. Ik gebruik pillen tegen hoofdpijn. De huisarts heeft uitgelegd dat ik hier beter mee kan stoppen. Zij heeft tips gegeven hoe ik zonder pillen met mijn hoofdpijn om kan gaan.
Tijdens mijn eerste zwangerschap dronk ik wel eens een glaasje wijn en rookte ik af en toe een sigaretje. De huisarts heeft mij uitgelegd waarom stoppen met roken en alcohol al voordat je zwanger bent belangrijk is. De huisarts heeft ook vragen gesteld over ziektes in de familie. Zij heeft uitgelegd dat deze ziektes niet gevaarlijk zijn voor de baby.

Wilt u naar het kinderwensspreekuur? Maak dan een afspraak!

U kunt een afspraak maken met de assistente van de huisarts. Aan het project is een onderzoek verbonden. Als u een afspraak maakt wordt u gevraagd of u bereid bent hieraan deel te nemen. Dit is echter niet verplicht.

Met vriendelijke groet uw huisarts

Stage S4: Stage 2: Second Pretest (Final Invitational Flyer)

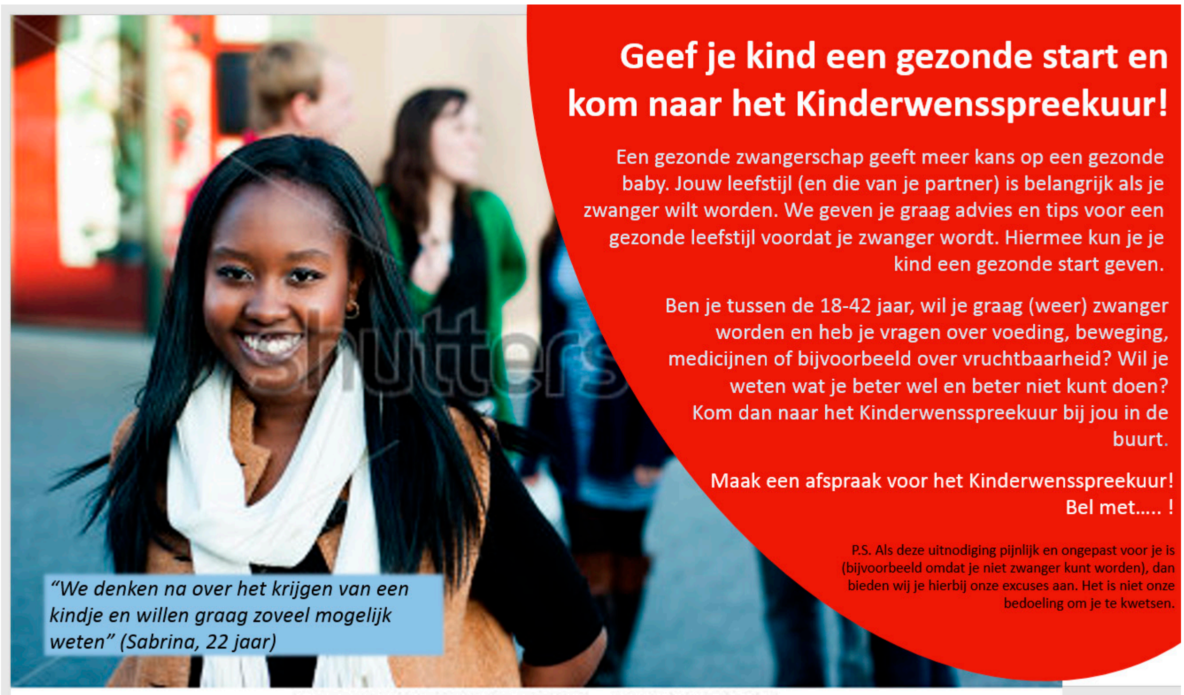

Supplement: Supplementary file 1 [file ijerph-16-04223-s001.pdf]
